# Homeless Services Data vs Health Records to Recognize Homelessness

**DOI:** 10.1001/jamahealthforum.2025.5328

**Published:** 2025-11-26

**Authors:** Mario A. Pita, C. Holland McDowell, Phillip Ma, Hanna Haile, Daniel Ludi, Niraj Gowda, Jillian S. Catalanotti

**Affiliations:** 1 The George Washington University School of Medicine and Health Sciences, Washington, DC; 2 Georgetown University School of Medicine, Washington, DC; 3 Weill Cornell Medicine, New York, New York; 4 University of Illinois Chicago College of Medicine, Chicago; 5 Emory University School of Medicine, Atlanta, Georgia

## Abstract

This cross-sectional study compared data from the Homeless Management Information System with electronic health records to quantify how often homelessness was potentially missed during emergency department visits without admission and inpatient admissions; and identify factors associated with misses.

## Introduction

People experiencing homelessness frequently use emergency departments (EDs) and hospitals,^1,2^ yet hospitals inconsistently screen for homelessness,^2,3^ limiting opportunities for patient-centered care and linkage to services at discharge. Measuring how often homelessness goes unrecognized is challenging. This cross-sectional study compared data from the Homeless Management Information System (HMIS) with electronic health records (EHRs) to (1) quantify how often homelessness was potentially missed during ED visits and inpatient admissions; and (2) identify factors associated with misses. Combining HMIS data with EHR review provides a uniquely comprehensive analysis of gaps in recognizing homelessness.

## Methods

George Washington University’s institutional review board approved this study and waived written informed consent. We used the Strengthening the Reporting of Observational Studies in Epidemiology (STROBE) reporting guidelines. Data were collected from 2021 to 2022 and analyzed in 2024.

HMIS contains data on all consumers of homeless services in Washington, DC. We selected a simple random sample of 10 000 *literally homeless* (as defined by the Department of Housing and Urban Development) adults in HMIS who used services between September 1, 2019, and February 29, 2020. HMIS provided identifiers, demographics, family status, and services used. We searched 1 hospital’s EHR for matches on name, birth date, and gender, and included individuals with 1 or more ED visit or admission in the same 6-month period.

Reviewers examined EHR documentation from the first ED visit and/or admission, including notes by clinicians and ancillary staff; discharge summaries; and *International Statistical Classification of Diseases and Related Health Problems, Tenth Revision (ICD-10)* codes for language indicating homelessness/unstable housing. Reviewers (M.A.P., C.H.M., H.H., D.L., and N.G.) coded homelessness as documented or missed. See eMethods in Supplement 1 for details.

We screened covariates with 2-sided χ^2^ test or logistic regression (age). Variables with *P* < .05 or conceptual importance entered multivariable logistic models. We fit 3 logistic models with identical covariates: ED only, admission only, and combined to compare odds of missed homelessness between settings. The combined model used mixed-effects specification with a patient random intercept so an ED visit and admission from the same patient were not treated as independent.

## Results

After excluding 161 HMIS records with incomplete identifiers, 4893 of 9839 clients (49.7%) matched to EHR charts (Table); 1145 patients met inclusion criteria (967 ED visits, 332 admissions), 813 of 1145 (71%) had no admissions. Homelessness was potentially missed in 615 ED visits (63.6%) and 130 admissions (39.2%). In the mixed-effects logistic model, the odds ratio for missed homeless during ED visits compared to admissions was 2.80 (95% CI, 2.07-3.76).

**Table.** Patient Characteristics and Univariable Associations With Missed Recognition of Homelessness in Emergency Department (ED) Visits and Inpatient Admissions

Variable	All participants, No. (%)^a^ [N = 1145]	ED visits without admission			Inpatient admissions		
		No. (%)^a^		*P* value	No. (%)^a^		*P* value
		Identified (n = 352)	Missed (n = 615)		Identified (n = 202)	Missed (n = 130)	
Age, mean (SD), y^b^	45.2 (14.5)	48.2 (13.9)	43.2 (14.1)	<.001	49.1 (15.1)	43.9 (15.3)	.004
Adult with family^c^	116 (10.1)	9 (2.6)	67 (10.9)	<.001	19 (9.4)	32 (24.6)	<.001
Services used							
Emergency shelter^d^	976 (85.2)	321 (91.2)	508 (82.6)	<.001	181 (89.6)	102 (78.5)	.005
Transitional housing	137 (12.0)	29 (8.2)	90 (14.6)	.004	16 (7.9)	16 (12.3)	.19
Race and ethnicity^e^							
Hispanic	43 (3.8)	13 (3.7)	19 (3.1)	.61	13 (6.4)	8 (6.2)	.92
Non-Hispanic Black	987 (86.2)	284 (80.7)	546 (88.8)	<.001	166 (82.2)	117 (90.0)	.05
Non-Hispanic White	82 (7.2)	41 (11.7)	35 (5.7)	<.001	19 (9.4)	2 (1.5)	.004
Insurance^f^							
Medicare	172 (15.0)	65 (18.5)	69 (11.2)	.002	44 (21.8)	24 (18.5)	.46
Medicaid	471 (41.1)	147 (41.8)	239 (38.9)	.38	118 (58.4)	55 (42.3)	.004
DC Alliance^g^	348 (30.4)	72 (20.5)	203 (33.0)	<.001	47 (23.3)	57 (43.9)	<.001
Commercial	41 (3.6)	18 (5.1)	19 (3.1)	.11	6 (3.0)	3 (2.3)	.72
Uninsured	229 (20.0)	93 (26.4)	131 (21.3)	.07	21 (10.4)	10 (7.7)	.41
Primary diagnosis							
Medicine	332 (29.0)	81 (23.0)	164 (26.7)	.21	90 (44.6)	52 (40.0)	.41
Psychiatric	134 (11.7)	70 (19.9)	22 (3.6)	<.001	54 (26.7)	10 (7.7)	<.001
Surgical or trauma	153 (13.4)	29 (8.2)	92 (15.0)	.002	16 (7.9)	26 (20.0)	.001
Gender^h^							
Female	455 (39.7)	141 (40.1)	231 (37.6)	.44	82 (40.6)	58 (44.6)	.47
Male	679 (59.3)	208 (59.1)	378 (61.5)	.47	117 (57.9)	72 (55.4)	.65

^a^ Percentages are column percentages.

^b^ Age is modeled as a continuous variable; *P* value reflects change in odds per 1-year increase by simple logistic regression; all other *P* values are from χ^2^ tests (2-sided).

^c^ Adult with family indicates an adult accompanied by 1 or more minor children as listed in the Homeless Management Information System. All other adults are listed as single.

^d^ Emergency shelter and transitional housing indicate service usage during the 6-month study window.

^e^ Race and ethnicity recorded as either Asian, American Indian/Alaska Native, Native Hawaiian/Other Pacific Islander, other, or unknown for 33 participants. These encounters are included in the overall denominators but are not shown separately because the small numbers preclude reliable percentage estimates.

^f^ Insurance categories add to greater than 100% because some participants had more than 1 type of health insurance.

^g^ DC Alliance is the District of Columbia Healthcare Alliance, a locally funded insurance program for low-income residents.

^h^ Gender recorded as transgender for 11 patients (9 ED visits and 3 admissions). These encounters are included in the overall denominators but are not shown separately because the small numbers preclude reliable percentage estimates.

For ED visits, younger age, non-Hispanic Black race, and being an adult with family were associated with higher odds of missed homelessness (Figure). For admissions, surgical or trauma diagnosis was associated with higher odds of missed homelessness; emergency-shelter use was associated with lower odds. Psychiatric primary diagnosis was associated with lower odds of missed homelessness in both settings.

**Figure.**
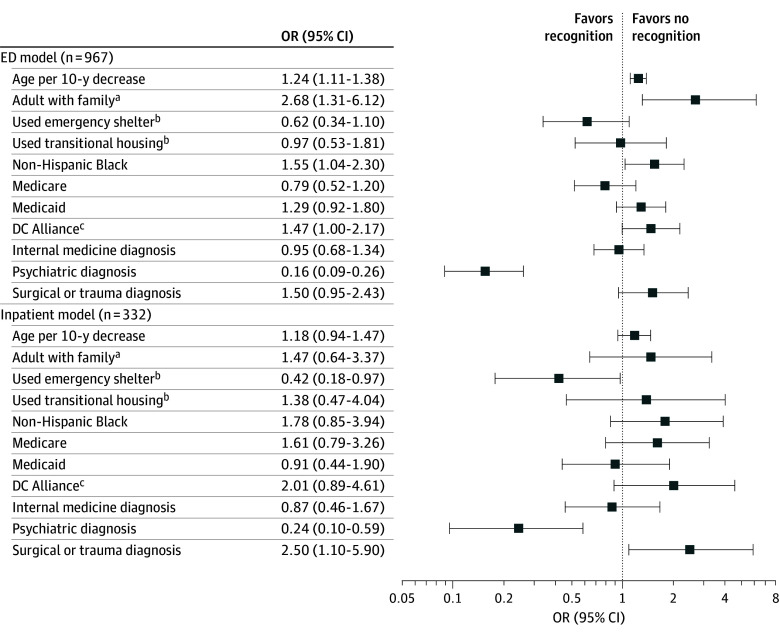
Adjusted Odds Ratios (ORs) for Missed Recognition of Homelessness During Emergency Department (ED) Visits and Inpatient Admissions Adjusted odds ratios (markers) and 95% CIs (horizontal lines) are plotted on a base-10 logarithmic scale. The dashed vertical line marks no association (odds ratio, 1.0). ^a^Adult with family indicates an adult accompanied by 1 or more minor children as listed in the Homeless Management Information System. ^b^Emergency Shelter and Transitional Housing indicate service usage during the 6-month study window. ^c^DC Alliance is the District of Columbia Healthcare Alliance, a locally funded insurance program for low-income residents.

## Discussion

Identifying patients as people experiencing homelessness allows clinicians to create patient-centered plans and refer to services at discharge. Since 2022, the Centers for Medicare & Medicaid Services requires screening for housing instability during admissions.^4^ We found homelessness was potentially missed in 39.2% of admissions, supporting universal screening using validated tools, like Veterans Affairs Homelessness Screening Clinical Reminder.^5^ Because ED visits were more frequent than admissions in our sample, screening and service linkage may have a greater impact if performed in EDs.

The odds of potentially missed homelessness were almost 3-fold higher for ED visits. Rapid, problem-focused ED care may limit comprehensive history-taking or documentation. Psychiatric diagnosis was associated with identifying homelessness. Explanations include exhibiting behaviors cueing screening for homelessness or extensive psychiatric history-taking.

Strengths of this study include HMIS verification of homelessness and extensive EHR review. Limitations include single institutional data and documentation as surrogate for identification. To minimize possibility of homelessness changing between EHR and HMIS entries, we studied literally homeless adults in a 6-month window.

HMIS and EHR comparison is feasible to quantify potentially missed homelessness. As EHRs increasingly incorporate referral to service organizations, consideration of bidirectional information sharing may be warranted.

## Notes

Supplement 1. eMethods eReferences

Supplement 2. Data Sharing Statement
