# Error in the Figure

**DOI:** 10.1001/jamahealthforum.2025.6677

**Published:** 2026-01-16

In the Research Letter titled “Homeless Services Data vs Health Records to Recognize Homelessness,”^1^ published online on November 26, 2025, there was an error in the labeling of the Figure. Odds ratio values less than 1 favor recognition, and values more than 1 favor no recognition. This article was corrected online.

## References

[1] Pita MA, McDowell CH, Ma P, . Homeless services data vs health records to recognize homelessness. JAMA Health Forum. 2025;6(11):e255328. doi:10.1001/jamahealthforum.2025.5328 41296374 PMC12658657

