# Supplementary material for: Homeless Services Data vs Health Records to Recognize Homelessness
Source: JAMA Health Forum. 2025 Nov 26;6(11):e255328. doi: 10.1001/jamahealthforum.2025.5328 (PMC12658657; doi:10.1001/jamahealthforum.2025.5328)
Supplement: Supplement 1. — eMethods eReferences [file jamahealthforum-e255328-s001.pdf]

Supplemental Online Content

Pita MA, McDowell HC, Ma P, et al. Homeless Services Data vs Health Records to Recognize Homelessness. *JAMA Health Forum*. 2025;6(11):e255328. doi:10.1001/jamahealthforum.2025.5328

eMethods

eReferences

This supplemental material has been provided by the authors to give readers additional information about their work.

© 2025 Pita MA et al. *JAMA Health Forum*.

eMethods

Setting and Participants:

This study was performed at a 395-bed urban academic hospital located in Washington, DC. In 2019, DC had three university hospitals, four community hospitals, one children's hospital and one Veterans Affairs (VA) hospital. Excluding data from the children's and VA hospitals, according to the District of Columbia Hospital Association, in 2019, the study hospital had 17.9% of DC's hospital beds (395/2205), performed 22.0% of all admissions (21,170/96,066), and performed 20.3% of all ED visits (70,775/34,9287).[1]

The Housing and Urban Development's annual Point-in-Time (PIT) Count reports the number of sheltered and unsheltered PEH on a single night in January. In 2019, PIT found 4915 adult PEH in DC.[2] Location data within DC are not publicly available. Detailed data regarding Emergency Department (ED) and hospital utilization by people experiencing homelessness (PEH) are not publicly available for DC, including their rates of visiting different hospitals. Our study sample of 1145 HMIS-matched patients cannot be directly correlated to those in the PIT count, but would represent approximately 23% of them, which is consistent with our hospital's share of ED visits and admissions, suggesting a reasonable sampling of PEH in DC.

Our study included only adults (≥18 years-old) in HMIS who were defined as "literally homeless" according to the U.S. Department of Housing and Urban Development definition, which denotes individuals who either sleep in a place not meant for habitation, live in a shelter, or currently have lived in an institution for ≤90 days immediately preceded by one of the first two scenarios.[3] We chose a 6-month window to minimize the possibility that a participant's

© 2025 Pita MA et al. *JAMA Health Forum*.

housing status may have changed between HMIS and EHR data entries. We intentionally chose a time-period before the COVID-19 pandemic because Washington, DC instituted “COVID hotels” for PEH, which may have impacted how individuals identified their own housing status and how health professionals viewed housing status.

Information in HMIS shared with the research team included name, date of birth and self-identified gender, race and ethnicity. Adults were categorized as being “with family” if they were accompanied by ≥1 minor child; all other adults were categorized as “single.” Services used included emergency shelter or transitional housing.

Chart Review Process:

For ED visits without admission, reviewers examined all major note types associated with the visit: physician/advanced practice provider (APP) notes, nursing notes, social work (SW) notes, and psychiatry consultation notes. For inpatient admissions, reviewers examined the Problem List section of the EHR for ICD-10 code Z59.0 = Homelessness; physician/APP admission note, last inpatient progress note and discharge summary; all SW notes; psychiatry consultation notes; and all physical/occupational therapy (PT/OT) notes. The Problem List pulls ICD-10 codes for diagnoses entered discretely at any time throughout the admission. Interim inpatient progress notes and routine inpatient nursing notes were not reviewed for admissions.

All reviewers were trained in a standardized procedure for chart review. Only pre-specified note types were reviewed (above). For each note, the first step was a series of five free-text searches for: “homeless”, “domicile” (to find “undomiciled” or “un-domiciled”), “shelter”, “street”, “housing” (to find “unstable housing”, “lacks housing”, etc.). If the note had no mention of these

© 2025 Pita MA et al. *JAMA Health Forum*.

words, then a full manual review of the note was performed. We found that the majority of notes with homeless documentation contained these words, which streamlined review. Reviewers abstracted insurance and primary diagnosis category.

For quality assurance, a senior reviewer independently re-abstracted 15 encounters per reviewer. Discordances were resolved by consensus, then five additional encounters per reviewer were re-abstracted to confirm concordance.

Supplement References:

1. District of Columbia Hospital Association. *Utilization Indicators: Calendar Year 2019*. Issued May 2020. Accessed August 23, 2025. https://dcha.org/data-publications/.
2. The Community Partnership for the Prevention of Homeless. *2019 Point-in-Time Count of Persons Experiencing Homeless in the District of Columbia*. Accessed August 23, 2025. https://dhs.dc.gov/sites/default/files/dc/sites/dhs/page_content/attachments/2019%2BDC%2BPIT%2BResults%20%281%29.pdf.
3. United States Department of Housing and Urban Development. *Category 1: Literally Homeless.* Accessed August 23, 2025. https://www.hudexchange.info/homelessness-assistance/coc-esg-virtual-binders/coc-esg-homeless-eligibility/four-categories/category-1/.

© 2025 Pita MA et al. *JAMA Health Forum*.
