# Supplementary material for: Homeless Services Data vs Health Records to Recognize Homelessness
Source: JAMA Health Forum. 2025 Nov 26;6(11):e255328. doi: 10.1001/jamahealthforum.2025.5328 (PMC12658657; doi:10.1001/jamahealthforum.2025.5328)
Supplement: Supplement 2. — Data Sharing Statement [file jamahealthforum-e255328-s002.pdf]

Data Sharing Statement

Pita. Homeless Services Data vs Health Records to Recognize Homelessness. *JAMA Health Forum*. Published November 26, 2025. doi:10.1001/jamahealthforum.2025.5328

Data
Data available: No

Additional Information
Explanation for why data not available: We received information about housing status via a Data Use Agreement with The Community Partnership, which keeps a repository of all individuals who utilize housing related social services in Washington, DC. Our Data Use Agreement does not include public sharing.
